# A longitudinal study of CPAP therapy for patients with chronic cough and obstructive sleep apnoea

**DOI:** 10.1186/1745-9974-9-19

**Published:** 2013-07-11

**Authors:** Krishna M Sundar, Sarah E Daly, Alika M Willis

**Affiliations:** 1 Department of Medicine, University of Utah, Salt Lake City, UT, USA; 2 Merrill Gappmayer Family Practice Clinic, Provo, UT, USA; 3 Division of Pulmonary, Critical Care & Sleep Medicine, University of Utah, Salt Lake City, UT 84132, USA

**Keywords:** Cough, Sleep apnea, Obstructive, Continuous positive airway pressure

## Abstract

**Background:**

Chronic cough patients are rendered therapies for gastro-esophageal reflux (GERD), upper airway cough syndrome (UACS) and cough-variant asthma (CVA) with varying benefit. Idiopathic or unexplained cough has emerged as an important clinical entity in both primary care and subspecialty clinics. Recent evidence points to a link between chronic cough and untreated obstructive sleep apnea (OSA).

**Methods:**

A prospective observational study was done to evaluate the effect of OSA therapy in patients with chronic cough. Patients enrolled into the study underwent questionnaires to evaluate for GERD, UACS and CVA along with screening questionnaires for OSA and daytime sleepiness. The Leicester cough questionnaire (LCQ) was done at baseline and during serial visits to evaluate cough intensity and was used as the primary outcome measure of the effect of CPAP therapy on chronic cough.

**Results:**

Out of 37 patients enrolled into the study, only 28 patients had follow up LCQ scores available and therefore underwent analysis. 22/28 patients were suspected to have OSA based on abnormal STOP-BANG screening questionnaire scores and overnight oximetry abnormalities. Of these 19/28 patients had overnight attended polysomnography with definitive diagnosis of OSA yielding a 68% prevalence of OSA in our chronic cough population. Chronic cough patients treated for OSA tended to be older with a significantly higher BMI than chronic cough patients without OSA. Significant improvement of LCQ scores occurred with CPAP therapy for OSA in chronic cough patients.

**Conclusion:**

OSA is significantly prevalent in chronic cough patients. Subjects with chronic cough and OSA tend to be older and obese. Treatment of OSA in chronic cough patients yields significant improvement in their health status.

## Background

Chronic cough is an important health-care problem in both primary care and subspecialty clinics [1]. The 2006 ACCP guidelines emphasize the need to aggressively address the etiologies of gastro-esophageal reflux disease (GERD), upper airway cough syndrome (UACS) and cough-variant asthma (CVA) while treating patients with chronic cough [2]. Despite undertaking prolonged courses of therapies directed at GERD, UACS and CVA, a significant proportion of patients continue to experience persisting cough [3]. The percentage of chronic unrelenting cough termed as “unexplained” or “idiopathic cough” has ranged up to 42% in different studies [4].

Newer approaches at dissecting the etiology of unexplained cough have focused upon the role of ongoing non-acid reflux [5], under-recognized vocal cord dysfunction [6] and untreated obstructive sleep apnea (OSA) [7] in perpetuating chronic cough. A large retrospective study from our clinic population during the period 2006–2009 revealed that 44% of the 75 patients with chronic cough had underlying OSA [8]. More importantly, therapy for comorbid OSA with continuous positive airway pressure (CPAP) resulted in improvement or resolution of cough in 93% of the patients [8]. While the prevalence of OSA in this large retrospective analysis was felt to be quite high, it was still considered to be an underestimate as all patients were not systematically screened for OSA [8]. Additionally, while a role for treatment of OSA was implicated in the resolution of cough, all patients received concurrent therapy for GERD, UACS or CVA that may have contributed to the resolution of chronic cough in this study [8].

The aim of the current study was to prospectively investigate the impact of CPAP therapy on predefined-cough measures in patients with OSA associated chronic cough. Patients referred primarily for chronic cough were evaluated for OSA using validated questionnaires and objective testing for OSA; the effect of CPAP on those diagnosed definitively with comorbid OSA was serially assessed. The primary outcome was the effect of CPAP therapy on the total Leicester cough questionnaire score.

## Methods

All consecutive patients with chronic cough seen at Intermountain Utah Valley Pulmonary Clinic, Provo, Utah were given the option to enroll into this study between March 2010- February 2012. Inclusion criteria included the following:

– Cough more than 2 months duration

– Normal spirometry and diffusion capacity <70% of predicted

– Normal chest radiographs and/or CT scans of the chest

– Age >18 years

Exclusion criteria included:

– History of lung disease in the form of prior diagnoses of asthma, COPD, interstitial lung disease or sarcoidosis.

– Chronic disease states such as congestive heart failure, chronic kidney disease, cancer, need for immunosuppressive therapy, or any debilitating illness that prevented follow up.

– Any history of smoking or history of being in occupations that resulted in inhalational exposures.

– Pregnancy.

– Use of opiate containing cough suppressants and/or first-generation antihistaminics.

Based on the above inclusion–exclusion criteria, 37 consecutive patients with chronic cough were enrolled over a 2-year period. A total of 46 patients were found eligible for the study but 9 patients either did not consent for the study, were on cough suppressants that made them ineligible, or turned out to have other diagnoses on follow-up (e.g. one patient had mediastinal adenopathy on chest CT). After enrolment, patients undertook the following questionnaires at baseline and on follow up –

1. Assessments for cough severity using the Leicester cough questionnaire (LCQ). LCQ is a validated, well-studied 19-point questionnaire that assesses the impact of cough severity on multiple aspects of daily living [9]. Amongst available cough questionnaires, LCQ has been shown to correlate the most with objective cough frequency as assessed by cough monitors [10]. In the current study, subjects underwent LCQ assessments at baseline and during each follow up visit.

2. Screening for OSA was done at baseline visit using the STOP-BANG questionnaire. Amongst available screening tools for OSA, the 8-point STOP-BANG questionnaire has emerged as one of the most easy to use [11]. While the sensitivity for a STOP-BANG score of 3 or more for detecting OSA (apnea-hypopnea index (AHI)>5/hour) is 83.6-85.1%, using a STOP-BANG score of 3 or more is not entirely reliable for excluding mild OSA [12]. Workup for further diagnosis of OSA was left up to the treating physician. Further evaluation for OSA was based upon a STOP-BANG score of ≥3 along with results of Epworth Sleepiness Score (ESS), and the finding of other symptoms relating to sleep-disordered breathing and daytime dysfunction. Many patients underwent screening oximetry before they were subjected to a polysomnography (PSG) for definitive diagnosis of OSA. Based upon the results of PSG, the OSA severity was categorized based upon the apnea-hypopnea index -AHI. Since the workup for OSA was left up to the treating physician, there was a potential for underestimation of the prevalence of OSA in chronic cough patients since all of these patients were not subjected to the gold-standard for diagnosing OSA – attended PSG. Attended PSG was done at an accredited sleep laboratory (Utah Sleep Disorders Center, Provo, Utah) and studies were performed according to American Academy of Sleep Medicine Criteria. The recording montage included EEG leads C4-M1, C3-M2, Cz-Oz, Cz-Fz, bilateral electrooculogram, chin EMG, electrocardiogram, a microphone for recording snoring, monitors of airflow, chest and abdominal effort recordings, oximetry, and if applied, the level of CPAP mask flow and leak. Sleep stage scoring was performed according to AASM criteria. Apnea was defined as a decrease to ≤10% of baseline of thermistor airflow signal and a hypopnea was defined as a 30% or greater decrease in airflow signal accompanied by a 4% oximetric desaturation or an arousal.

3. Patient-reported assessments of symptoms of GERD, UACS and CVA at baseline. This was done using validated questionnaires for each of these commonly-treated chronic cough etiologies using the GERD [13], SNOT-20 [14], and asthma life questionnaires [15]. Each of these questionnaires is used for screening and for following the severity of symptoms of GERD, UACS and asthma over time [13-15]. Usage of these validated questionnaires was felt to be necessary as clinician-driven assessments of GERD, UACS and CVA in chronic cough patients tend to be subjective with insufficient documentation regarding symptomatology of these problems in chronic cough patients. The GERD questionnaire comprised of 4 questions relating to symptoms of GERD (or their control) with grading of intensity of these symptom intensity from 0–3 (score range 0–12 [13]). The SNOT-20 involved responses to 20 questions with a symptom response score of 0–5 (score range 0–100) [14] and the asthma life questionnaire has 20 questions with a yes or no response (score range 0–20) [15].

Following baseline assessments, additional workup and management of OSA was left up to the treating pulmonologist. Since patients were often on therapies for UACS, GERD and CVA at the time of initial evaluation, the need for continuation of these therapies was left up to the treating physician. While it was recommended that newer therapies for GERD, UACS or CVA would not be started unless felt absolutely necessary, in order to ensure patient comfort, treating physicians were allowed to start additional treatments for GERD, CVA and UACS if felt absolutely essential. The use of cough-suppressants such as first-generation antihistaminics and opiates was disallowed due to the possibility that these treatments would confound effects on chronic cough from OSA therapy. Therefore patients receiving these therapies were excluded from the study.

Follow up after initial baseline visit was done at the discretion of the treating physician. Patients non-compliant with CPAP therapy or PSG recommendations were continued till the end of the study if they continued to complete their LCQs during follow up visits. The study and approval for publication without disclosure of identifying data was obtained from the Intermountain Institutional Review Board, Intermountain Healthcare, Salt Lake City, Utah.

## Results

**Figure 1:**
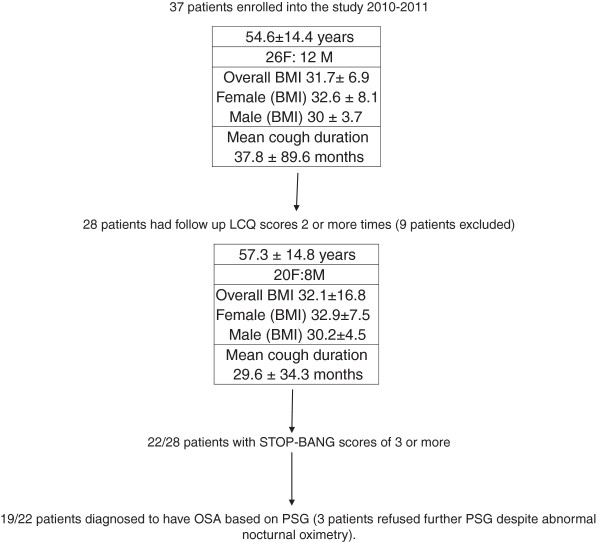
Flowchart detailing enrolment and follow up in the study.

Out of a total of 37 patients with chronic cough that were initially enrolled into the study between 2010–2012, 19 patients with chronic cough and objectively verified OSA were followed and showed significant improvement in LCQ scores following CPAP intervention. 9/37 patients enrolled were excluded due to non-compliance with follow up. Out of the 28 patients that completed follow-up LCQ questionnaires, 22 patients reported a STOP-BANG score of 3 or more leading to further evaluations for OSA (Figure 1). In these 22 patients, 13 oximetries were carried out all of which were abnormal. Out of these 22 patients, 3 patients were noncompliant with recommendations for further OSA evaluation but continued to follow-up on therapies for GERD (3/3) and UACS (1/3) (Figure 1). 19/28 patients had objective polysomnographic evidence of OSA. Out of these 19 patients, 2 patients had previously known OSA but were noncompliant with therapy and were optimized on CPAP based on prior polysomnographic data; 17 patients underwent PSGs following enrolment into the study. Of the 17 patients that were studied with PSG following enrolment into study, the mean AHI was 35.3±29.5 with 6/17 patients being in the mild OSA category (AHI 5-15/hr), 4/17 patients in the moderate OSA category (AHI 15-30/hr) and 7/17 patients being in the severe OSA category (AHI>30/hr).

**Figure 2:**
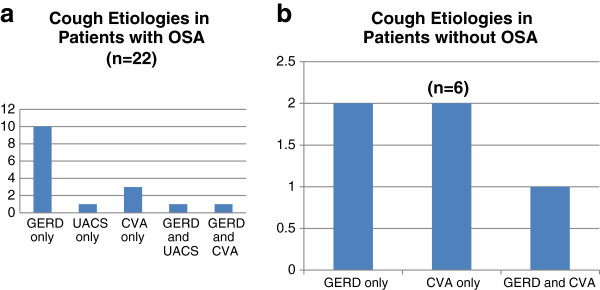
**(A &B) Etiologies of cough in patients with OSA and without OSA.**

15/19 patients that were treated with CPAP were on proton-pump inhibitors for GERD, 11/19 were on therapy for UACS and 7 were on treatment for CVA (Figure 2a). No ACE-inhibitor use was noted in this population. As reflected in the questionnaires obtained at time of enrolment for this study, mean symptom scores for GERD, UACS and asthma were low for the enrolled chronic cough subjects.

Table 1 details the clinical characteristics of the groups of patients followed in this study. Patients that were treated for OSA with CPAP were tended to be older, significantly heavier with higher STOP-BANG OSA screening scores. Although they tended to have longer durations of cough as compared to those diagnosed without OSA, there was no significant difference in durations of cough between patients with and without OSA (Table 1). The six patients that did not undergo further evaluation for OSA based on STOP-BANG scores and clinical evaluation comprised a small comparison group for the group of 19 patients that were treated with CPAP (Table 1). Interestingly, there was no significant difference in the mean scores on the GERD, SNOT-20 and ALQ questionnaires (Table 1) between the OSA and non-OSA groups although there were differences in cough therapies rendered for these two groups (Figure 2b).

**Table 1:** Results of baseline questionnaires between CPAP-treated and non-treated groups

	**Patients treated with CPAP (n=19)**	**Patients treated without CPAP (n=6)**	**P value**
Age (years)	58.7±15.1	44.7±13	***0.05***
Sex ratio	13 F:6 M	5 F:1 M	NA
BMI	34.9±6.7	26.5±4	**0.002**
Cough duration (months)	33.8±38.1	15.5±12.2	0.08
SNOT-20 score	8.8±4.2	5.5±3.1	0.8
GERD score	1.8±1.3	1±1.3	0.2
Asthma life questionnaire	38.8±20.5	36.3±19.3	0.07
STOP-BANG score	4.3±1.7	1.5±2.7	**0.009**
0-2	1/19	4/6	
3-5	14/19	2/6	
5-8	4/19	0/6	
Epworth Sleepiness score	11.25±4.2	4±4.8	**0.01**

Abbreviations: *CPAP* Continuous positive airway pressure, *BMI* Body mass index, *SNOT* Sino-nasal outcome test, *GERD* Gastroesophageal reflux disease.

**Figure 3:**
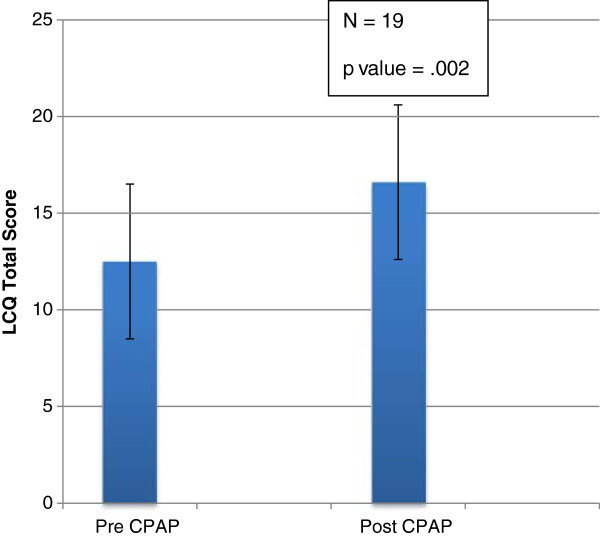
Change in LCQ total score with CPAP therapy.

Table 2 shows changes in total LCQ scores for the CPAP-treated group of chronic cough patients with breakdown of LCQ scores into different domains. In patients treated with CPAP, there was a significant improvement in total LCQ scores, and LCQ scores in psychological and social domains (Table 2 & Figure 3).

**Table 2:** Leicester cough questionnaire scores change in CPAP-treated chronic cough patients

	**CPAP treated patients (n=19)**		
**LCQ domains**	**Baseline**	**Final**	**P value**
Physical	4.6±1.2	5.2±1.1	0.086
Pyschologic	4.1±1.4	5.3±1.2	**0.005**
Social	3.8±1.7	5.5±1.6	**0.003**
LCQ Total	12.5±4	16.6±3.9	**0.002**
Duration of follow up (days)	157.3±108.5		

Abbreviations: *CPAP* Continuous positive airway pressure, *LCQ* Leicester cough questionnaire.

## Discussion

The current emphasis on finding mechanistic bases of cough have led to the recognition that chronic cough is a multi-trigger driven process that is not amenable to a single therapeutic intervention [16,17]. A disease with a potential to affect number of processes within the upper and lower airways is OSA [18]. In order to understand the effects from an intervention on chronic cough, studies in the last decade have typically used validated cough questionnaires, cough sensitivity assessments or objective cough monitoring to assess cough serially [19]. Amongst these, serial LCQ measurement of total and individual domain scores in chronic cough subjects is the most-validated and widely utilized instrument to assess effects from an intervention [19] and a change in LCQ score(s) was used to assess the effect of CPAP therapy in chronic cough patients with co-morbid OSA.

Based on the results of the STOP-BANG screening questionnaire and the more definitive gold standard of attended PSG, this study shows a high prevalence of obstructive sleep apnea in chronic cough patients. Reasons for the high prevalence of OSA noted in this study are manifold. OSA is a commonly-encountered disorder that has reached epidemic proportions [20]. Screening questionnaires while useful in directing the need for further testing however are not 100% sensitive for detecting milder forms of disease. Therefore while these questionnaires are useful for elaborating the need for further evaluation for sleep-disordered breathing, in themselves their results have to be used in conjunction with other diagnostic modalities for OSA [12]. In our study, while we used a cut-off of a score of 3 or more on the STOP-BANG score as a way of directing need for further testing of OSA, not all physicians used this solely for ordering further polysomnographic testing. Increasing STOP-BANG scores are correlated with higher likelihood for OSA and all patients with scores of 5 or more were tested for OSA and all these patients were found to have significant OSA on PSG. 1/19 patients in the OSA group had STOP-BANG scores of less than 3 and 2/6 patients in the non-OSA group had STOP-BANG scores of 3–4. There was also wide variation in the ESS scores in OSA vs. non-OSA groups although OSA patients had a significantly increased ESS than the non-OSA group (Table 1).

The gold standard for OSA diagnosis remains the attended PSG. In our study 22/28 patients were felt to have a high likelihood of OSA based the combination of abnormal STOP-BANG scores and overnight oximetries yielding an OSA prevalence of 78% in chronic cough patients. Based on definitive PSGs, 19/28 patients had OSA yielding a prevalence of 68% OSA in this chronic cough population. This high prevalence of OSA in our chronic cough population may have been from higher BMIs of the patient population in this study [8]. This finding of increased BMIs in our population contrasts with the average BMI reported by recent studies on chronic cough from Europe [5,21]. Aside from a significant difference in BMIs in OSA and non-OSA groups, there was also a trend towards a lower age in chronic cough patients without OSA. Chronic cough patients without OSA tended to be younger with a higher female preponderance. These findings are in congruence with the results of studies that have shown a correlation between OSA prevalence and age [22], OSA and male sex [22], and OSA and BMI [23].

A number of questionnaires were administered in this study that assessed symptom intensity scores for commonly encountered entities of GERD, UACS and CVA. While the scores from these questionnaires do not accurately assess the severity of the individual disorders of GERD, UACS and CVA, this was undertaken in order to compare the OSA and non-OSA groups in terms of the prevalence for symptoms of these three commonly encountered disorders. While the therapies for GERD, UACS and to some extent even CVA is somewhat empiric in most chronic cough patients, a greater problem lies in the lack of documentation of symptoms pertaining to these three conditions in a consistent manner by physicians. Therefore instead of trying to decipher underlying cough etiologies based upon treatments rendered and clinic records, we decided to use these validated questionnaires to develop scores for GERD, UACS and CVA. This approach to categorize and evaluate these three conditions in diagnostic and testing protocols has been used to direct comprehensive therapy for chronic cough patients [24,25] although given the high prevalence of these conditions in the general population, their relation to patient’s cough is unclear [26] . In this study as well, there appeared to be a preponderance of therapies rendered for GERD as well as therapies for multiple etiologies for cough as was noted in our retrospective study [8]. Even though patients with chronic cough and OSA tended to be older and significantly heavier, there was no difference in patient reported scores for UACS, GERD and CVA between OSA and non-OSA subjects..

In the subjects treated with CPAP, there was a significant improvement in LCQ scores in chronic cough subjects with OSA. This occurred in both the total LCQ scores and the individual psychological and social domains. This supports prior observations that CPAP therapy for comorbid OSA improves cough outcomes in this population [8]. LCQ change is an established method for measuring efficacy of interventions on cough [27-29] and not all studies have included a placebo arm while assessing efficacy of an intervention on LCQ improvement [30]. In these chronic cough studies, the mean LCQ change with intervention have ranged from 2.1- 3.5 and with placebo 1.1-2.6 [27-29]. One of the problems with our study was that even though a significant change was not expected in the LCQ score of those patients that were not treated with CPAP for OSA, the three non-compliant patients in the study also showed an increase in LCQ scores. This underscores the need for studying the effect of CPAP on cough intensity in chronic cough patients using a placebo arm.

Recently the observation that CPAP therapy can improve cough in patients with concomitant OSA has been reported by others [31,32]. In one case report, an improvement in Ryan score (a measure of pharyngeal pH done through a transnasal probe) occurred following CPAP therapy accompanied by improvement in cough intensity and sensitivity [31]. Further studies need to not only clarify cough populations that improve following CPAP therapy but also the mechanistic bases of cough improvement after CPAP therapy. Studies using a placebo arm (sham CPAP controls) are needed to understand fully the benefit accorded by CPAP therapy on cough intensity and sensitivity in patients with chronic cough.

## Conclusion

There is a high prevalence of OSA in patients with chronic cough. Patients with chronic cough and OSA tend to be older and significantly heavier. Significant improvement in health status occurs following CPAP therapy occurs in chronic cough patients with OSA. Further studies trying to demonstrate a role for CPAP therapy should include a placebo arm (or sham CPAP) for conclusively establishing a benefit of CPAP therapy on chronic cough. The mechanisms for cough improvement from CPAP therapy can multiple and future studies should also attempt to understand the pathways involved in cough improvement with CPAP therapy.

## Abbreviations

LCQ: Leicester cough questionnaire; OSA: Obstructive sleep apnea; GERD: Gastroesophageal reflux disease; UACS: Upper airway cough syndrome; CVA: Cough variant asthma; BMI: Body mass index; PSG: Polysomnography; CPAP: Continuous positive airway pressure.

## Competing interests

The authors declare that they do not have any financial competing interests in relation to the current manuscript.

## Authors’ contributions

KS – study design, conduct, data analysis and write-up. SD – study design, conduct, data collection and analysis. AW – study conduct, data collection and analysis. All authors read and approved the final manuscript.
